# Metabolic engineering of *Corynebacterium glutamicum* for anthocyanin production

**DOI:** 10.1186/s12934-018-0990-z

**Published:** 2018-09-14

**Authors:** Jian Zha, Ying Zang, Matthew Mattozzi, Jens Plassmeier, Mamta Gupta, Xia Wu, Sonya Clarkson, Mattheos A. G. Koffas

**Affiliations:** 1 Department of Chemical and Biological Engineering, Center for Biotechnology and Interdisciplinary Studies, Rensselaer Polytechnic Institute, Troy, NY 12180 USA; 2 College of Forestry, Nanjing Forestry University, Nanjing, 210037 China; 3 Conagen Inc., Bedford, MA 01730 USA; 4 BASF Corporation, Tarrytown, NY 10591 USA; 5 Department of Environmental Sciences, DAV University, Jalandhar, Punjab 144 001 India

**Keywords:** Anthocyanin, *Corynebacterium glutamicum*, Catechin, Flavonoid, UDP-glucose

## Abstract

**Background:**

Anthocyanins such as cyanidin 3-*O*-glucoside (C3G) have wide applications in industry as food colorants. Their current production heavily relies on extraction from plant tissues. Development of a sustainable method to produce anthocyanins is of considerable interest for industrial use. Previously, *E. coli*-based microbial production of anthocyanins has been investigated extensively. However, safety concerns on *E. coli* call for the adoption of a safe production host. In the present study, a GRAS bacterium, *Corynebacterium glutamicum*, was introduced as the host strain to synthesize C3G. We adopted stepwise metabolic engineering strategies to improve the production titer of C3G.

**Results:**

Anthocyanidin synthase (ANS) from *Petunia hybrida* and 3-*O*-glucosyltransferase (3GT) from *Arabidopsis thaliana* were coexpressed in *C. glutamicum* ATCC 13032 to drive the conversion from catechin to C3G. Optimized expression of *ANS* and *3GT* improved the C3G titer by 1- to 15-fold. Further process optimization and improvement of UDP-glucose availability led to ~ 40 mg/L C3G production, representing a > 100-fold titer increase compared to production in the un-engineered, un-optimized starting strain.

**Conclusions:**

For the first time, we successfully achieved the production of the specialty anthocyanin C3G from the comparatively inexpensive flavonoid precursor catechin in *C. glutamicum*. This study opens up more possibility of *C. glutamicum* as a host microbe for the biosynthesis of useful and value-added natural compounds.

**Electronic supplementary material:**

The online version of this article (10.1186/s12934-018-0990-z) contains supplementary material, which is available to authorized users.

## Background

**Fig. 1:**
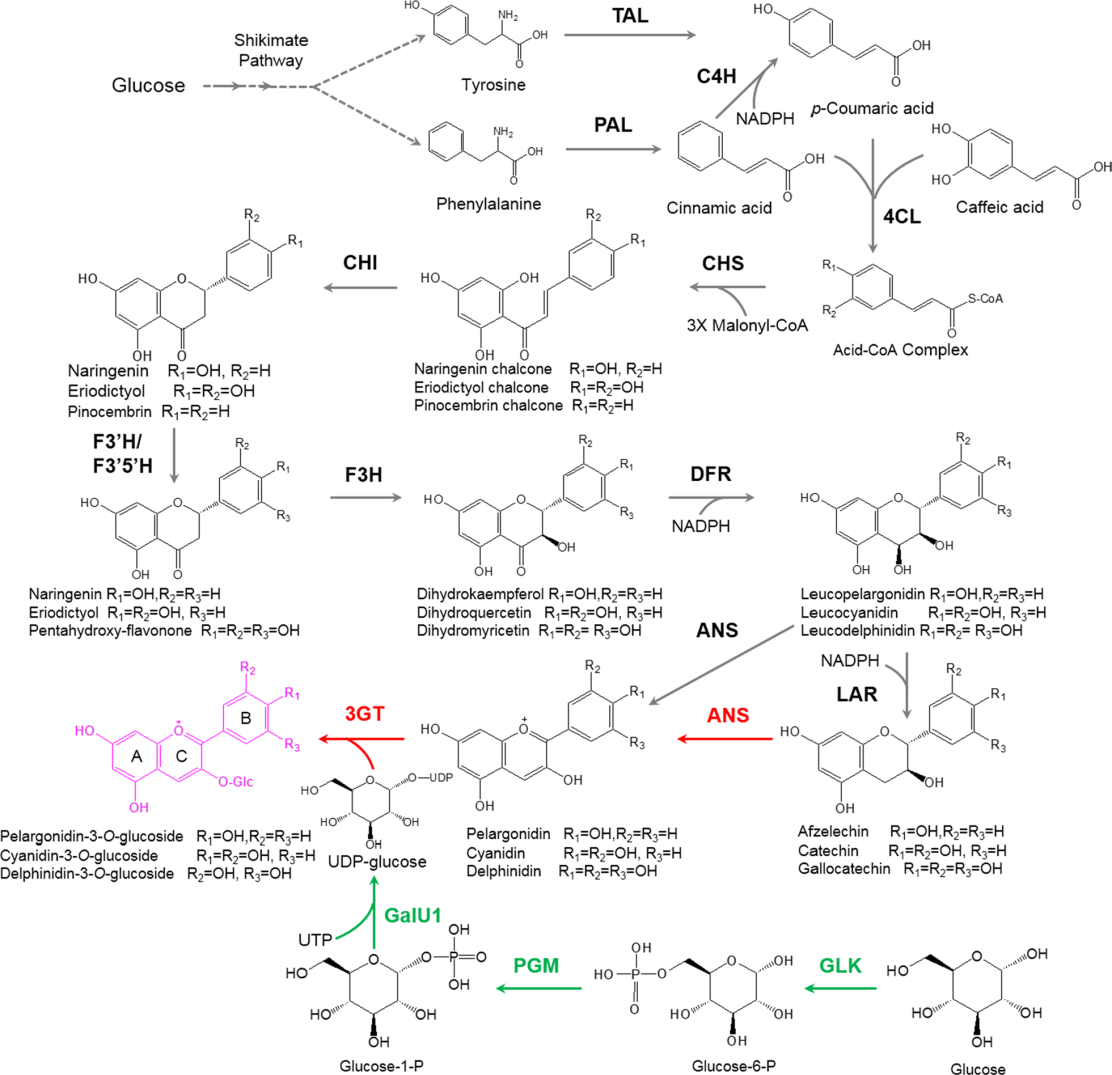
Biosynthetic pathway of anthocyanins (labeled in pink) in plants and the introduced pathway for cyanidin 3-*O*-glucoside production from catechin in *C. glutamicum*. The introduced pathway consisting of ANS and 3GT is highlighted in red, and the biosynthetic pathway of UDP-glucose in *C. glutamicum* is marked in green. Abbreviations of the enzymes in the pathway: *PAL* phenyl ammonia lyase, *TAL* tyrosine ammonia lyase, *C4H* cinnamate 4-hydroxylase, *4CL* 4-coumaroyl-CoA ligase, *CHS* chalcone synthase, *CHI* chalcone isomerase, *F3′H* flavonoid 3′-hydroxylase, *F3′5′H* flavonoid 3′,5′-hydroxylase, *F3H* flavanone 3-hydroxylase, *DFR* dihydroflavonol 4-reductase, *LAR* leucocyanidin reductase, *ANS* anthocyanidin synthase, *3GT* flavonoid 3-*O*-glucosyltransferase, *GLK* glucokinase, *PGM* phosphoglucomutase, *GalU1* UTP-glucose-1-phosphate uridylyltransferase

Anthocyanins are valuable flavonoids that have diverse applications in food processing, cosmetic production, and nutraceutical manufacturing [1–3]. They are synthesized via the general flavonoid pathway, which converts tyrosine or phenylalanine to the flavonoid precursor flavanones, such as naringenin (Fig. 1). Hydroxylation of these flavanones at ring B and reduction by dihydroflavonol 4-reductase (DFR) form leucoanthocyanidins. These compounds, or their reduced form flavan-3-ols through the action of leucoanthocyanidin reductase (LAR), can be further oxidized by anthocyanidin synthase (ANS) for the generation of the unstable flavylium cation anthocyanins, which are then linked to a glucosyl residue at C3 in ring C to form anthocyanin-3-*O*-glucosides such as cyanidin-3-*O*-glucoside (C3G). Other modifications such as glycosylation at other hydroxyl groups, methylation, hydroxylation, and acylation on the ring skeleton result in diverse anthocyanin molecules [4–6].

At present, anthocyanins used in industry are mainly obtained by extraction from plant tissues, which is subject to seasonal supply and quality control concerns inherent in agriculture [7, 8]. An alternative way of production is biosynthesis in metabolically engineered microorganisms, attributed to some advantages of microbes over plants, including ease of cultivation and fast growth, availability of sophisticated genetic tools, and well-defined metabolic networks and models. The most commonly used chassis microbe in metabolic engineering is *E. coli*, which has been extensively engineered for the biosynthesis of several natural flavonoids such as naringenin, kaempferol, and quercetin [9–11]. *Saccharomyces cerevisiae* and *Streptomyces venezuelae* have also been deployed for natural flavonoid production [12–14].

The biosynthesis of anthocyanins has been conducted in microorganisms for over a decade. In 2005, Yan et al. cloned and expressed in *E. coli* the genes of flavanone 3-hydroxylase (*F3H*) and *ANS* from *Malus domestica*, *DFR* from *Anthurium andraeanum*, and flavonoid 3-*O*-glucosyltransferase (*F3GT*) from *Petunia hybrida* [15]. The recombinant strain produced 6.0 μg/L of C3G and 5.6 μg/L pelargonidin 3-*O*-glucoside using naringenin and eriodictyol as the respective precursors. Subsequent selection of plant-derived gene orthologs, optimization of UDP-glucose pool, regulation of precursor uptake and optimization of the production process dramatically enhanced production of pelargonidin 3-*O*-glucoside and C3G, with their titers reaching 113 mg/L and 350 mg/L, using afzelechin and catechin precursors, respectively [16–18]. Recently, de novo production of ~ 10 mg/L pelargonidin 3-*O*-glucoside from glucose has been achieved via an *E. coli* consortium. In this study, the first node strain was a highly efficient tyrosine producer and the entire pathway from tyrosine to pelargonidin 3-*O*-glucoside was split into four strains [19]. However, all the reported recombinant hosts producing anthocyanins are currently limited to *E. coli* derivatives.

*Corynebacterium glutamicum*, having been widely used in industrial production of amino acids such as L-glutamate and l-lysine [20, 21], is advantageous over other bacteria in several aspects: (1) it does not produce endotoxins like *E. coli* and is generally regarded as safe for the production of pharmaceuticals, food and cosmetics; (2) it has been broadly applied in industry, and current facilities can be retrofitted to produce chemicals of interest; (3) its metabolism can be easily rewired for target compounds through the readily available genetic tools and metabolic models [22–25]. Recently, this strain has been successfully engineered to produce flavanones or stilbenes by expressing CHS and CHI or stilbene synthase, respectively [26]. Subsequently, the heterologous pathways introduced into *C. glutamicum* have been extended to flavonols (such as kaempferol and quercetin) and pterostilbene [27].

In this study, we constructed recombinant *C. glutamicum* strains that could produce the anthocyanin C3G from catechin. Through optimization of gene parts, expression levels, fermentation process parameters, and supply of the cosubstrate UDP-glucose, the engineered strain was able to produce ~ 40 mg/L C3G from 500 mg/L of catechin. To the best of our knowledge, this is the first report of biosynthesis of any anthocyanin in *C. glutamicum*, and this study further potentiates *C. glutamicum* for its application in flavonoid bioproduction.

## Results

### Optimization of *3GT* expression for C3G production in *C. glutamicum*

**Fig. 2:**
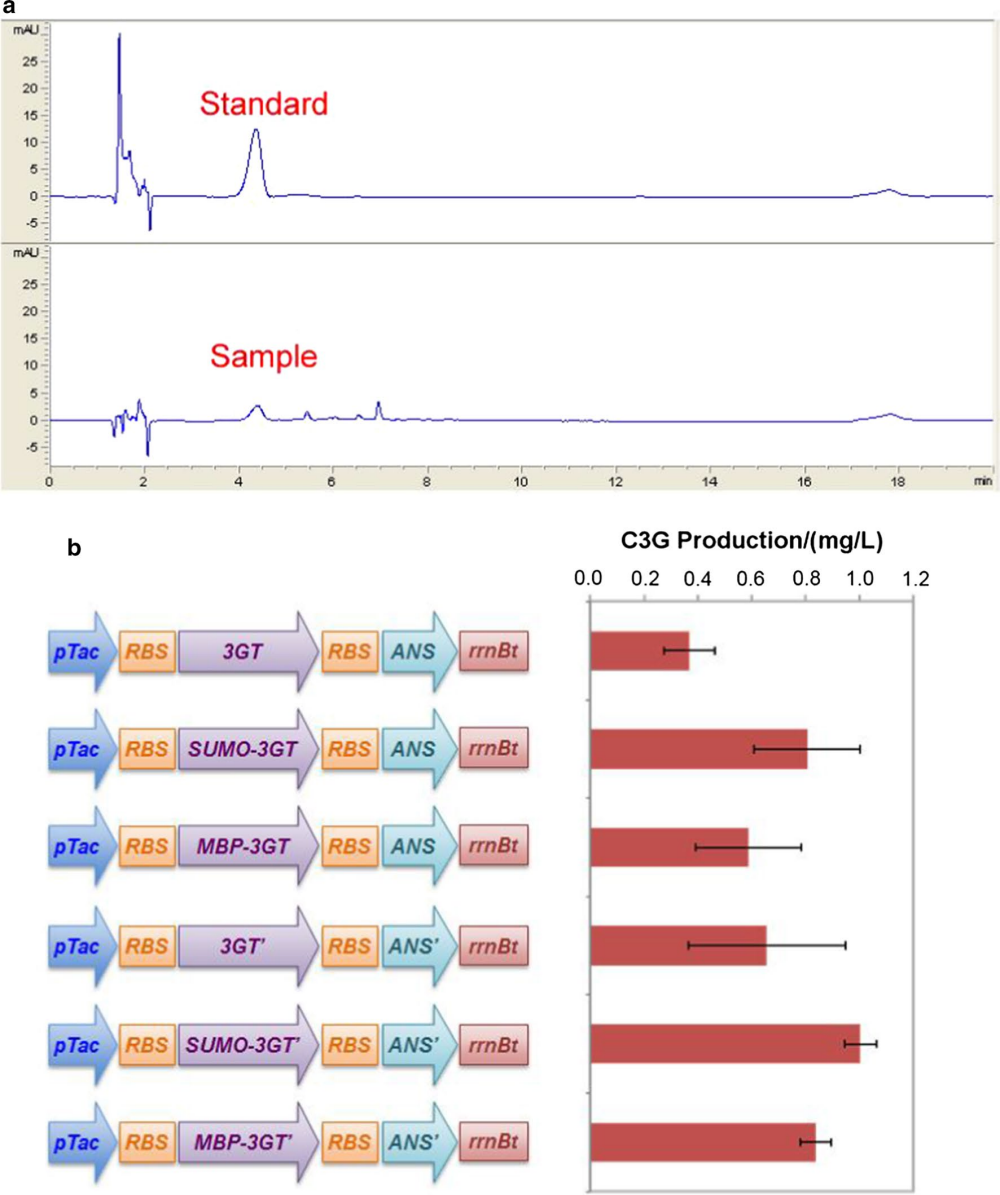
C3G production by recombinant strains harboring six different modules of variant *3GT* and *ANS* in an operon form. **a** The HPLC chromatograms of the standard C3G and C3G produced from the recombinant strain expressing wildtype *ANS* and *3GT*; **b** the genetic contexts of the six constructs and their C3G production. *ANS’* and *3GT’* indicate codon optimized genes. The data represents mean ± standard deviation of three independent experiments

In our previous research, *3GT* was found to be partially expressed as insoluble inclusion bodies in *E. coli*, leading to a very low yield of the functional enzyme [28]. A universal approach to increase the soluble expression of heterologous proteins is through the fusion of a protein or peptide tag, which is highly soluble in the host strain even at a very high expression level, such as maltose-binding protein (MBP) and small ubiquitin-like modifier (SUMO) [29, 30]. Here, we fused genetically either MBP or SUMO to the N-terminus of 3GT. In addition, considering the plant origin of *3GT* and its possible inefficient translation in microbes, we optimized the codon according to *C. glutamicum* codon preference. These modifications resulted in six recombinant strains (Fig. 2), and their C3G producing capabilities were evaluated in the synthetic medium CGXII, which is commonly used for *C. glutamicum* cultivation and fermentation. However, none of these strains could produce C3G. The CGXII medium, while containing all the essential nutrients to support fast cell growth and metabolism, might not contain enough components required for successful expression of the anthocyanin pathway, especially the genes *ANS* and *3GT*, as indicated by the low expression level from SDS-PAGE analysis (Additional file 1: Figure S1). Based on this assumption, another medium AMM, suitable for anthocyanin biosynthesis in *E. coli* [19], was tested with a slight change in the amount of supplemented biotin (modified AMM). Interestingly, all the *C. glutamicum* strains could synthesize C3G in this medium (Fig. 2a, Additional file 1: Figure S2). This phenomenon has also been observed in the production of flavan-3-ols and anthocyanins in *E. coli*, where the minimal medium M9 proved to be better than the rich medium LB [15, 31]. Although the C3G yields were below 1 mg/L (Fig. 2b) for all the constructs, it is clear that fusion of SUMO and MBP alone improved the production by 110% and 58%, respectively; codon optimization led to a 71% increase in titer for the wildtype genes, and 25% and 42% increase for SUMO and MBP fusion, respectively. The less pronounced effect of codon optimization on the enzymes with fusion tags was probably due to already enhanced gene expression.

**Fig. 3:**
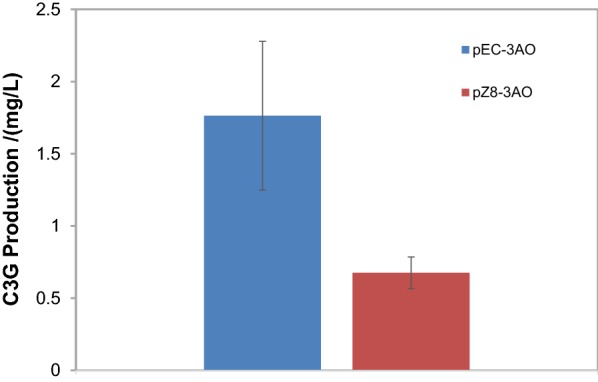
C3G production in *C. glutamicum* strains expressing the chimeric fusion of 3GT and ANS (3AO). The chimeric enzyme was expressed in the plasmid pEC-XK99E (pEC-3AO) and pZ8-1 (pZ8-3AO) for inducible and constitutive expression of 3AO, respectively

A possible cause of the low titer of C3G formation is that anthocyanidin, the catalytic product of ANS in the anthocyanin pathway, was unstable under the culture condition and was degraded before it could be further converted by 3GT. To test this possibility, the strategy of fusion expression of the two genes was adopted, which has been proposed to facilitate the formation of a protein complex, to increase the local concentration of the unstable reaction intermediate, and to accelerate the overall conversion [16]. An enzyme chimera 3AO, with 3GT fused to the N-terminus of ANS, was constructed genetically and expressed in plasmids pEC-XK99E and pZ8-1, which supported inducible and constitutive expression of 3AO, respectively. While both types of 3AO expression increased C3G formation in modified AMM, the inducible expression helped to reach a titer of 1.7 mg/L (Fig. 3), which was 3.6-fold higher compared to the unfused expression of the wildtype genes.

### Enhanced expression of *ANS* improves C3G production

**Fig. 4:**
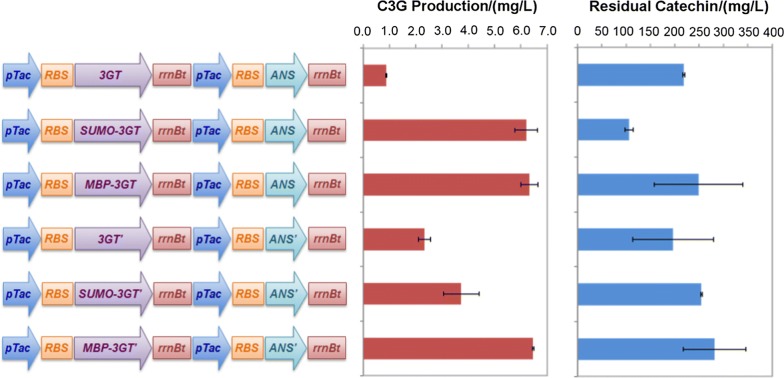
C3G formation and catechin consumption in recombinant *C. glutamicum* strains containing monocistronically constructed wildtype and codon-optimized (indicated by the prime symbol) *3GT* and *ANS* with different fusion tags. The data represents mean ± standard deviation of three independent experiments

In all the tested gene constructs, the expression of *3GT* and *ANS* was in the operon or fusion form and under the control of a single promoter, resulting in coupled expression. Hence, limited transcription of *3GT* could lead to compromised expression of *ANS*. To uncouple the expression of the two genes, monocistronic gene constructs were generated with the insertion of an *rrnB* terminator, a *tac* promoter, and a ribosome binding site (RBS, AAAGGAGGA) between the genes encoding 3GT and ANS (Fig. 4). The resulting strains produced 1.3- to 9.6-fold more C3G in modified AMM compared with those carrying the corresponding operon constructs. Among these, the strain harboring wildtype *ANS* and *3GT* with SUMO fusion (*SUMO*-*AG*) displayed the strongest catechin consumption with the highest C3G formation (> 6 mg/L) (Fig. 4), which was 6.6-fold more than the strain expressing coupled *SUMO*-*3GT* and *ANS*, and 2.5-fold higher than the strain with *3AO* expression. This indicates that the SUMO-AG strain has the best capability of channeling catechin to cyanidin, although cyanidin did not accumulate due to its instability under the present condition. It is interesting to note that cells expressing *MBP*-*AG* (wildtype *ANS* and *3GT* with the MBP tag) and *MBP*-*A′G′* (MBP fusion of codon-optimized *3GT* and *ANS*) produced similar amount of C3G with less substrate compared to SUMO-AG-expressing strain. Since the substrate itself is unstable, its fast utilization is of great importance. As a result, the strain expressing *SUMO*-*AG* was selected for the subsequent genetic optimization.

### Comparison of different promoters for C3G pathway gene expression

Gene expression in *C. glutamicum* was slower than that in *E. coli* (Additional file 1: Figure S3) [32]. Thus it is possible that the expression of C3G pathway genes could be rate limiting, leading to the accumulation of the substrate catechin, which is unstable in the medium. Hence, faster and stronger expression of *ANS* or *3GT* might elevate C3G production. In all the above gene constructs, the expression was controlled by the *tac* promoter. Being a strong promoter, P_tac_ is inducible and only initiates gene expression in the presence of an inducer. To further enhance gene expression, we chose two strong constitutive promoters, *eftu* and *sod*, which are widely employed in *C. glutamicum* to allow for continuous expression throughout the life cycle of the cells and to increase metabolic flux towards the desired metabolites. With such durable gene expression, there could be more molecules of the active enzymes available for the biotransformation process. To this end, we placed *SUMO*-*3GT* and *ANS* each under the control of either P_sod_ or P_eftu_, generating four combinations (S–S, S–E, E–S, and E–E), and examined C3G production of the *C. glutamicum* strains with these promoter combinations. A higher C3G yield was obtained with the *eftu* promoter than with its P_sod_ counterpart; when both genes were controlled by P_eftu_, the yield was > 2-fold higher than the S–S combination (Table 1). In addition, the effect of P_eftu_ was more noticeable on *3GT* than on *ANS*. However, when compared with the *tac* promoter, these constitutive promoters reduced C3G formation by 52–84%, indicating that the strong constitutive expression of 3GT and ANS is not suitable for the functional operation of the pathway in the host. Therefore, the *tac* promoter was used in subsequent studies.

**Table 1:** Production of C3G by different engineered strains with combinations of constitutive promoters

Strain	Plasmid included	C3G titer (mg/L)
S–S	pZM1-sodSUMO-3GT-sodANS	1.02 ± 0.09
S–E	pZM1-sodSUMO-3GT-eftuANS	1.20 ± 0.11
E–E	pZM1-eftuSUMO-3GT-eftuANS	3.13 ± 0.02
E–S	pZM1-eftuSUMO-3GT-sodANS	2.68 ± 0.01

### Regulation of UDP-glucose supply for improved C3G production

**Fig. 5:**
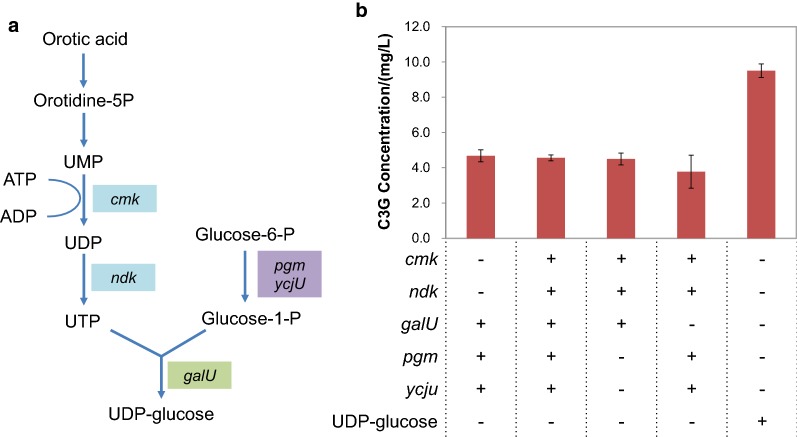
The effect of UDP-glucose biosynthesis modules on C3G production. **a** The metabolic pathway of UDP-glucose biosynthesis from orotic acid in *E. coli*. **b** C3G production by different constructs expressing different pathway genes of UDP-glucose biosynthesis and with the addition of 10 mM UDP-glucose (control)

UDP-glucose takes part in the glycosylation of cyanidin in C3G biosynthesis and is regarded as an essential cosubstrate. Being a limiting factor in the formation of anthocyanins in *E. coli* [16, 17], UDP-glucose could also have a critical impact on C3G production in *C. glutamicum*. The general strategies to increase the availability of UDP-glucose include amplification of the biosynthesis genes and blocking of the competitive UDP-glucose consumption pathways. To enhance the supply of internal UDP-glucose and to circumvent the tight regulation on UDP-glucose biosynthesis imposed by native regulatory networks, the UDP-glucose biosynthesis pathway from *E. coli*, consisting of genes *cmk*, *ndk*, *galU*, *pgm* and *ycjU*, was heterologously expressed in the anthocyanin-producing strain (SUMO-AG) (Fig. 5). To investigate the diverse effect of these genes in UDP-glucose synthesis and C3G production, different combinations of pathway genes were generated and expressed along with *ANS* and *SUMO*-*3GT*. However, the expression of these modules did not help to increase C3G titer (Fig. 5). This is inconsistent with previous studies that heterologous expression of *galU* alone from *E. coli* conferred improved supply of UDP-glucose and enhanced biosynthesis of trehalose and glycogen to *C. glutamicum* [33, 34]. A possible explanation for the lack of a positive effect in our system could be that the multiple genes from *E. coli* were not able to coordinate with the native UDP-glucose biosynthesis network for UDP-glucose accumulation, inducing negative responses such as translational imbalance [35]. The expression of *E. coli* UDP-glucose pathway might also activate the degradation of UDP-glucose, as in the case of glycogen or trehalose biosynthesis in *C. glutamicum*.

**Fig. 6:**
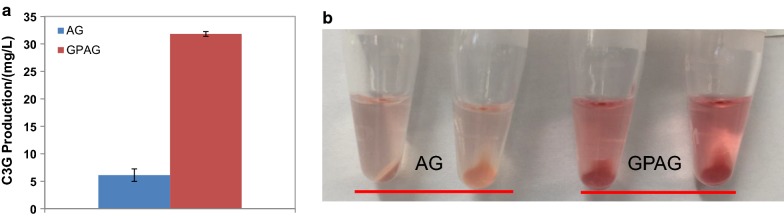
C3G production by *C. glutamicum* strain GPAG. The strain was grown in AMM supplemented with 0.2 mg/L biotin. IPTG (1.0 mM) and catechin (500 mg/L) were added 6 h post inoculation as indicated by an arrow. **a** C3G production from strains AG and GPAG. **b** Extracted supernatants of 48-h fermentation cultures from strains AG (left) and GPAG (right)

A different route to an abundant supply of UDP-glucose is to amplify endogenous pathway genes. Previous studies have shown that overexpression of genes *pgm* (cg2800) and *galU1* (cg1004), both essential in the UDP-glucose biosynthesis pathway [36], increases the level of UDP-glucose for cyanidin glycosylation [16, 37]. Therefore, we constructed *C. glutamicum* strain GPAG overexpressing both genes upon IPTG induction, and observed 4.2-fold higher C3G production (reaching 31.8 mg/L) (Fig. 6a) using the same fermentation process. At the same time, culture supernatants turned pinker after acidification compared with the parent strain AG (Fig. 6b).

### C3G production using optimized fermentation conditions

**Fig. 7:**
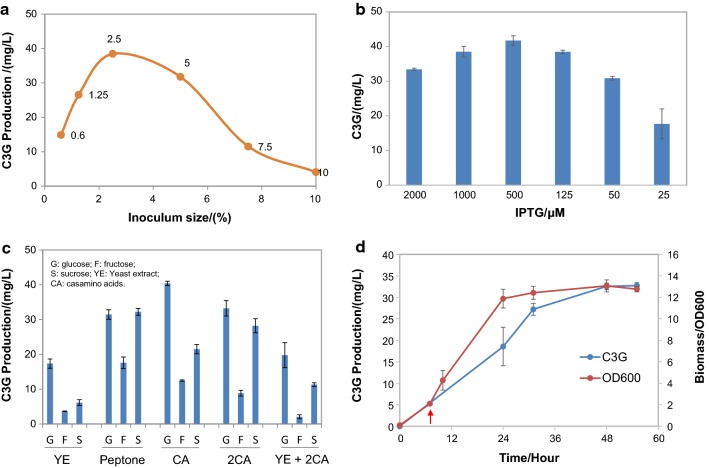
Bioprocess optimization for improved C3G production on **a** inoculum size, **b** IPTG concentration, and **c** selection of carbon sources (20 g/L of glucose, fructose, or sucrose) and nitrogen sources (2 g/L yeast extract, 2 g/L peptone, 2 or 4 g/L casamino acids (termed CA or 2CA), or 4 g/L casamino acids plus 14 g/L yeast extract). **d** C3G production in flasks using optimized fermentation conditions. The arrow indicates induction by 0.5 mM IPTG

Process optimization is indispensible in microbial fermentation for optimal production of the target metabolites. Among the many parameters in the fermentation process, the inoculum size, the inducer concentration, and the medium composition are some of the most basic and important factors, and their influence on C3G formation was investigated in engineered *C. glutamicum* strain GPAG. As shown in Fig. 7a, an increasing inoculum size first rapidly improved C3G yield and then led to a compromised production. Since the induction time point was fixed as 6 h post sub-culture, a slightly higher inoculum size means a higher density and vitality of the cells; however, when the inoculum size reached a certain point, the cell density at the induction time could be very high and the cells could be in late log phase or stationary phase with lower metabolic activity, which might be unfavorable for the anthocyanin pathway gene expression. Thus, an inoculum ratio of 2.5% was used in the following fermentation process.

When the concentration of the inducer IPTG was studied for its impact on C3G generation, a trend similar to that of inoculum size was observed, with 0.5 mM IPTG induction increasing C3G yield by ~ 10% compared to induction by 1 mM IPTG (Fig. 7b). The selection of carbon and nitrogen sources also played a significant role in C3G bioconversion. Supplementation of 2 g/L casamino acids [38] did not considerably alter C3G yield, whereas 4 g/L casamino acids slightly reduced production, and yeast extract and peptone largely inhibited C3G generation. For all the tested nitrogen sources, a concomitant addition of a carbon source had the same pattern of impact, with glucose > sucrose > fructose in supporting C3G formation, except when peptone was used as the nitrogen source (Fig. 7c). Based on these observations, the optimal bioprocess for C3G production was established as 2.5% inoculum size with 500 μM IPTG induction at 6 h after sub-culture, in modified AMM medium supplemented with 20 g/L glucose and 2 g/L casamino acids, with the maximal C3G titer reaching 41.7 mg/L. To evaluate the fermentation performance in shake flasks, the same strain was tested using the optimized fermentation conditions, and a titer of ~ 33 mg/L was observed in flasks at 48 h post induction. Additionally, we carried out C3G production using whole cells as a biocatalyst and obtained 43.7 mg/L of C3G (Additional file 1: Table S3), which was slightly higher than that obtained from the growing cells. In the whole cell biotransformation, C3G was only produced when cells were resuspended in AMM (pH7.0), suggesting that the C3G pathway might be sensitive to pH, salts, and/or certain nutrients.

## Discussion

Microbial production of anthocyanins may be used as a feasible way of producing anthocyanins for research and industrial applications. Previously, efficient *E. coli*-based bioproduction of anthocyanins have been successfully achieved in our lab [16, 39]. However, the host strain *E. coli* contains some unfavorable intrinsic properties such as production of endotoxins and underlying pathogenicity. Thus, in the present study, we referred to a new host strain, *C. glutamicum*, for the production of C3G. Through a series of engineering and optimization, ~ 40 mg/L C3G was generated by the modified strain. Recently, employment of *C. glutamicum* as the host strain to produce some flavanones has been performed [26]. These attempts demonstrate that *C. glutamicum* is a possible choice of microbial production of flavonoids.

In engineered microbes, the heterologous expression of plant-derived genes is generally challenging, and issues such as incorrect protein folding and formation of inclusion bodies lead to poor production of functional enzymes involved in the metabolic pathways. Codon optimization and fusion expression are commonly used strategies to partially solve these problems [40–42]. In this study, we found that codon optimization of *ANS* and *3GT* had very limited positive effects on improving C3G production, indicating that the codon usage in *C. glutamicum* might fit well with that in plants for these two genes. In another study of flavanone pathway expression in *C. glutamicum*, however, codon-optimization is indispensable [26]. Fusion expression with tags are known to improve soluble expression of alien proteins in common host bacteria. In the present study, MBP and SUMO fusion led to enhanced C3G biosynthesis, suggesting better expression of 3GT in its active form. Another study also benefited from this method, in which the enzyme *cis*-itaconate decarboxylase with an MBP fusion had > 2-fold higher activity, and the fusion enzyme led to one-fold increase of itaconate production in *C. glutamicum* [43].

Besides the expression level of each individual gene, the ratio of expression levels of *3GT* and *ANS* was also found to be critical for anthocyanin production in *C. glutamicum*. ANS is pivotal in the biosynthesis of anthocyanins in plants, as a direct correlation between ANS expression and anthocyanin accumulation has been observed in fruits such as bilberries and apples [44, 45]. More transcripts of the *ANS* gene lead to more copies of the ANS enzyme, which can help to convert more catechin to cyanidin. In this study, the expression level of *3GT* decreased in the monocistronic form due to shortened translation time compared to that in the operon organization [46], resulting in a lower ratio of *3GT* and *ANS* expression levels. Thus, a balanced expression of *3GT* and *ANS* was achieved in the monocistronic form of pathway architecture, which could lead to enhanced C3G production as extensively demonstrated in metabolic engineering [47, 48].

Although sufficient expression of *ANS* and *3GT* was necessary for better generation of C3G in *C. glutamicum*, extremely strong expression did not translate to a higher yield. The same negative effect was again observed in our study of polyglutamic acid production in *C. glutamicum*, in which the *sod*-controlled pathway expression led to less efficient biocatalysis than the *tac*-controlled expression (unpublished data). This was unexpected because these strong promoters have been widely used in the production of amino acids and other chemicals [49–51]; and in naringenin production in *S. cerevisiae*, constitutive expression of pathway genes driven by strong promoters (such as *TDH3*) resulted in a much higher titer compared with gene expression driven by weak inducible promoters (*GAL1* and *GAL10*) [52, 53]. A possible explanation is that *eftu* and *sod* promoters are not applicable in the production of secondary metabolites in *C. glutamicum*. Given that the transcript threshold of *ANS* and *3GT* tolerated by the host cells may be much lower than that of the pathway genes in amino acid biosynthesis, particularly strong expression of *ANS* or *3GT* driven by promoter *sod* or *eftu* could bring severe metabolic burden to cells, thus leading to imbalanced metabolic pathways and limited generation of cofactors and cosubstrates. In this sense, moderate expression of both genes is crucial for high-titer production of anthocyanins, as in the case of resveratrol production in *E. coli*, in which modest constitutive expression (*gap* promoter), instead of strong inducible expression (T7 promoter), of *4CL* and the stilbene synthase gene led to a higher yield [54]. It could be inferred that optimal expression of flavonoid biosynthesis genes depends on the host strains, and the suitable expression configuration varies among strains and systems.

In the present study, the maximal conversion yield based on consumed catechin was ~ 30%, equivalent to the yield in *E. coli* [16]. In our preliminary test, the substrate catechin was shown to be stable in the growing culture. Thus, it could be postulated that the consumed catechin was converted to cyanidin. Given that cyanidin is very unstable at neutral pH, and that an obvious cyanidin peak was not detected in HPLC analysis, it can be inferred that the fast degradation of cyanidin is a possible limiting factor in C3G production. It should be noted that C3G is also unstable at neutral pH [55]; thus, C3G stabilization is important for its biosynthesis. This has been achieved in *E. coli* by conducting the biocatalysis at a low pH (e.g., pH 5.0) [16]. However, such a strategy was not feasible for *C. glutamicum* due to its high sensitivity to low pHs (Additional file 1: Table S3). Adaptation of *C. glutamicum* for better tolerance to low pHs could be a possible solution to improve C3G production in an acidic environment.

Apart from the modification on the anthocyanin pathway and stability of cyanidin and C3G, the supply of UDP-glucose is one of the most important factors in derteming C3G production. The intracellualr UDP-glucose is relatively stable and strictly controlled, with limited flow towards the formation of glycosylated anthocyanidin (C3G) as has been extensively demonstrated in the production of anthocaynins and other glycosylated flavonoids in *E. coli* [17, 56]. In the present study, coexpression of *pgm* and *galU1* increased the production of C3G, indicating that their expression could channel more glucose-6-phosphate to UDP-glucose. This strategy could be used in *C. glutamicum*-based biosynthesis of other UDP-glucose derived products, such as glycogen, glycosylated proteins, and sophorolipids. In addition, inhibition of UDP-glucose degradation pathways through gene knockout or CRISPR interference could be conducted to improve the accumulation of UDP-glucose and further elevate C3G production [57, 58].

## Conclusions

We have demonstrated the successful production of C3G in *C. glutamicum* from the comparatively abundant and inexpensive catechin. Through controlled regulation of the expression of the plant-derived anthocyanin pathway genes (*ANS* and *3GT*), fine-tuned supply of UDP-glucose, and optimized fermentation process, C3G titer was elevated from ~ 0.37 mg/L to ~ 40 mg/L, representing > 100-fold improvement. This is the first report of anthocyanin bioproduction in *C. glutamicum*, and opens up new possibilities of microbial production of flavonoids by the GRAS strain *C. glutamicum* beyond *E. coli*. The inter-correlation of the flavonoid pathway with aromatic amino acid production pathway, and the extensive application of *C. glutamicum* in industrial production of amino acids make this bacterium promising for high-titer flavonoid biosynthesis from inexpensive feedstocks. So far, the production of naringenin from extracellular tyrosine has been achieved in *C. glutamicum* [26], and high titer production of tyrosine (26 g/L) from glucose in *C. glutamicum* has been well established [59, 60]. Based on these advances, it could be anticipated that de novo production of C3G from cheap carbon sources such as glucose or sucrose by a single recombinant *C. glutamicum* or a mixed culture of *C. glutamicum* strains can be fulfilled in the near future [19, 61].

## Methods

### Bacterial strains and media

The strains used in the study are listed in Additional file 1: Table S1. *E. coli* DH5α was used for cloning and plasmid propagation, and was grown in Luria Broth (LB) medium (Sigma) supplemented with 50 mg/L kanamycin when necessary; agar (Sigma) was added to 15 g/L for the preparation of medium-agar plates. *C. glutamicum* ATCC 13032 was used as the host for flavonoid production in this study. *C. glutamicum* cells were generally grown in Brain Heart Infusion (BHI) medium (BD) and kept in BHI with glycerol (20%, v/v) at − 80 °C for long-term storage. Fermentation by *C. glutamicum* was conducted in AMM medium supplemented with 0.2 mg/L biotin [62]. AMM medium contained (per liter): glucose, 20 g; KH_2_PO_4_, 3.5 g; K_2_HPO_4_, 5.0 g; (NH_4_)_2_HPO_4_, 3.5 g; casamino acids, 2 g; MgSO_4_, 0.12 g; CaCl_2_, 11 mg; thiamine HCl, 0.5 mg; MOPS, 8.37 g; Tricine, 0.72 g; FeSO_4_·7H_2_O, 2.8 mg; NaCl, 2.92 g; NH_4_Cl, 0.51 g; MgCl_2_ 0.11 g; K_2_SO_4_ 0.05 g; and micronutrient mix ((NH_4_)_6_Mo_7_O_24_, 0.4 μg; H_3_BO_3_, 2.5 μg; CuSO_4_, 0.24 μg, MnCl_2_, 1.6 μg; and ZnSO_4_, 0.28 μg).

### Plasmid construction

The plasmids and primers used in the present study are listed in Additional file 1: Tables S1 and S2. The *ANS* gene from *Petunia hybrida* and 3GT from *Arabidopsis thaliana* were acquired through PCR amplification (ACCUZYME 2X mix, Bioline) using the plasmid pETM6-At3GT-m-PhANS in the Koffas lab [39]. Similarly, maltose-binding protein (MBP) tag or small ubiquitin-like modifier (SUMO) tag was amplified using the plasmid pMAL-c2X-PhANS [39] or pET His6 SUMO TEV LIC cloning vector (Addgene plasmid 29711). The codon-optimized genes of *ANS* and *3GT* were synthesized by Integrated DNA technologies (IDT, USA). The fusion of MBP or SUMO tag with wildtype or codon-optimized *3GT* genes was achieved by overlap extension PCR.

To construct expression plasmids of operon configurations, different versions of *3GT* genes were first inserted into pEC-XK99E using traditional restriction enzyme-based cloning, followed by insertion of the *ANS* gene. Expression plasmids with *ANS* and *3GT* in a monocistronic form were constructed by insertion of a fragment, consisting of *rrnB* terminator and a *tac* promoter, into plasmids of operon configurations described above. To obtain the plasmids expressing the fused gene of *3GT* and *ANS* (termed *3AO*) in *C. glutamicum*, *3AO* was amplified using the plasmid pCDF-3AO as the template and subsequently cloned into the expression plasmids pEC-XK99E and pZ8-1 by *EcoR*I and *Sal*I, respectively.

Other expression plasmids were built on the basis of pZM1 (to be published separately), which was created from the plasmid pZ8-Ptac (Addgene plasmid 740694) along the principle in the construction of ePathBrick vector pETM6 [63]. The genes in the UDP-glucose biosynthesis pathway in *E. coli* (*cmk*, *ndk*, *galU*, *pgm* and *ycjU*) or in *C. glutamicum* (*galU1* and *pgm*) were amplified from the genomic DNA of BL21 Star (DE3) or *C. glutamicum* ATCC 13032, which was extracted by PureLink Genomic DNA Kit (Invitrogen). Each gene was then cloned into pZM1 and assembled in a monocistronic form using a previously published method [63].

### Construction of recombinant *C. glutamicum* strains

A single colony of wildtype *C. glutamicum* ATCC 13032 was inoculated into 3 mL of BHI medium and grown at 30 °C and 225 rpm. After overnight growth, 2 mL culture was transferred to 50 mL fresh BHI medium and grown to OD_600_ of ~ 1.75. Cells were chilled on ice for 10 min and centrifuged for 5 min at 3500 rpm and 4 °C. The pellet was washed once with 50 mL of ice-cold 10% (v/v) glycerol containing 1 mM Tris (pH 7.5) in ultrapure water and once with 50 mL of ice-cold 10% (v/v) glycerol, and was then resuspended in 1 mL ice-cold 10% glycerol. Aliquots (100 µL) were stored at − 80 °C. For electroporation, cells were thawed on ice (10 min), mixed with ~ 100 ng plasmid, and transferred to an electroporation cuvette (2 mm gap). Electroporation was performed with an electroporator (Bio-Rad) at 25 μF, 200 W and 2.5 kV, yielding a pulse duration of ~ 5 ms. Immediately after electroporation, cells were mixed with 1 mL pre-warmed BHI in the cuvette, and were transferred to a 2-mL microcentrifuge tube. Cells were heat-shocked at 46 °C for 6 min in a water bath, transferred to a 14-mL culture tube (VWR), incubated for 2 h at 30 °C, and plated on LB-agar plates containing 25 mg/L kanamycin. Positive clones were validated by colony PCR, plasmid miniprep, and gene sequencing (Genewiz).

### Fermentation conditions

Glycerol stocks were streaked onto LB agar plates with 25 mg/L kanamycin. Single colonies were inoculated into 3 mL of BHI medium with 25 mg/L kanamycin in a 14-mL culture tube for overnight growth at 30 °C and 225 rpm. Fresh AMM (1 mL) with 25 μg/mL kanamycin in a single well of a polypropylene deep 48-well plate (5 mL, VWR) was inoculated with 25 μL of the overnight culture, or other volumes when noted. In the process of optimization of carbon and nitrogen sources in AMM, 20 g/L of glucose, fructose or sucrose as well as different nitrogen sources (2 g/L yeast extract or peptone, 2 or 4 g/L casamino acids, or 4 g/L casamino acids plus 14 g/L yeast extract [38]) was used to prepare AMM and to test their effect on C3G production. The culture was then incubated at 30 °C and 225 rpm for 6 h. IPTG and catechin (prepared as a 50 g/L stock solution in dimethylformamide: ethanol = 8:2, v/v) were added to final concentrations of 1 mM and 500 mg/L, respectively. Necessary supplements (2-oxoglutarate, 0.1 mM; sodium ascorbate, 2.5 mM; orotic acid, 0.1 mM) were also added from 50-fold concentrated stock solutions (for strains containing constitutive version of C3G module, catechin and supplements were fed at the beginning of the subculture). The culture was further grown for 24 h at 30 °C and 225 rpm, and then mixed with equal volume of acidified methanol (with 1% hydrochloric acid, v/v), followed by brief vertexing. Following centrifugation at 21,000×*g* for 10 min, the supernatant was used for subsequent HPLC analysis. Scaled-up fermentation was carried out similarly in a 125-mL PYREX Erlenmeyer Flask containing 15 mL fermentation medium. Three biological replicates were used in all experiments.

### Metabolite analysis

The supernatants of cell extracts were analyzed by a previously established method [39]. Briefly, 25 µL of each sample was loaded into Agilent 1200 series HPLC consisting of a ZORBAXSB-18 column (5 μm, 150 mm × 4.6 mm) and a diode array detector, and was separated by solvent A (0.1% formic acid in water) and solvent B (0.1% formic acid in acetonitrile) with a linear gradient change (10–40% B at 0–10 min and 40–60% B at 10–15 min) at 1 mL/min flow rate. Absorbance at 280 nm and 520 nm was monitored. Peak areas were calculated for concentrations of the relevant compounds using standards of catechin (Sigma) and C3G (Alkemist Labs). Student’s t test was used for statistical analysis.

### LC–MS analysis

Agilent 1200 series HPLC equipped with an Eclipse XDB-C18 column (5 μm, 150 mm × 4.6 mm) and an LTQ-ORBITRAP XL mass spectrometer was used. HPLC analysis was performed with solvent A (0.1% formic acid in water) and solvent B (0.1% formic acid in acetonitrile) at a flow rate of 250 µL/min with a linear gradient (5% B at 0–5 min, 5–45% B at 5–40 min, 45–90% B at 40–45 min, 90% B at 45–49.9 min, 90–5% B at 49.9–50 min, and 5% B at 50–60 min). Mass spectrometer was operated in a positive ion mode with 2-ppm mass accuracy. Mass spectra were acquired at a resolution of 60,000 in a detection range of M/Z 100–700. Acquisition parameters were set as follows: spray voltage 4.5 kV, capillary voltage 44 V, tube lens voltage 150 V, capillary temperature 250 °C, sheath flow rate 25, and auxiliary gas flow rate 5.

## Additional file

**Additional file 1: Figure S1.** SDS-PAGE (10% gel) analysis of proteins ANS and 3GT by recombinant *C. glutamicum* expressing *ANS* and *3GT* in various media. The *E. coli* strain expressing *ANS* and *3GT* was used as a positive control. *E. coli* was cultivated in AMM with 2% glucose and induced by 1 mM IPTG for 4 h before harvested for protein extraction. The *C. glutamicum* strain was grown in BHIS, AMM or CGXII medium and was induced by 1 mM IPTG at mid-exponential phase for 12 h. Red arrows indicate bands for ANS (48.5 kD) and 3GT (50.5 kD) (their molecular weight is too close, and only one merged band could be seen in the gel). **Figure S2.** Mass Spectrum identification of C3G in the fermentation products of recombinant *C. glutamicum* strains. TIC (A) and EIC (B) for mass range of C3G of the standard C3G; TIC (C) and EIC (D)for mass range of C3G for the fermentation products; Mass spectrum for C3G peak in the standard (E) and the fermentation products (F). **Figure S3.** Time course study of mCherry expression in *C. glutamicum*. The mCherry gene was cloned into the plasmid pZM1, and the expression of mCherry was indicated by the fluorescence intensity at an excitation wavelength of 588 nm and an emission wavelength of 618 nm. **Table S1.** Plasmids and strains used in the present study. **Table S2.** Primers used in this study. **Table S3.** C3G production using concentrated cells in different conditions. Cells grown in AMM (pH7.0) were induced with IPTG (0.5 mM) for 6 h and harvested. Then 5 ml of cells were resuspended in 1 ml of different buffers with 500 mg/L catechin and necessary supplements, including citrate buffer, potassium phosphate buffer, AMM (pH 5.0) and AMM (pH 7.0), respectively. The conversion process was conducted at 30 °C and 220 rpm for 48 h.
