# Supplementary material for: Metabolic engineering of Corynebacterium glutamicum for anthocyanin production
Source: Microb Cell Fact. 2018 Sep 14;17:143. doi: 10.1186/s12934-018-0990-z (PMC6138892; doi:10.1186/s12934-018-0990-z)
Supplement: Supplementary file 1 — Additional file 1: Figure S1. SDS-PAGE (10% gel) analysis of proteins ANS and 3GT by recombinant C. glutamicum expressing ANS and 3GT in various media. The E. coli strain expressing ANS and 3GT was used as a positive control. E. coli was cultivated in AMM with 2% glucose and induced by 1 mM IPTG for 4 h before harvested for protein extraction. The C. glutamicum strain was grown in BHIS, AMM or CGXII medium and was induced by 1 mM IPTG at mid-exponential phase for 12 h. Red arrows indicate bands for ANS (48.5 kD) and 3GT (50.5 kD) (their molecular weight is too close, and only one merged band could be seen in the gel). Figure S2. Mass Spectrum identification of C3G in the fermentation products of recombinant C. glutamicum strains. TIC (A) and EIC (B) for mass range of C3G of the standard C3G; TIC (C) and EIC (D)for mass range of C3G for the fermentation products; Mass spectrum for C3G peak in the standard (E) and the fermentation products (F). Figure S3. Time course study of mCherry expression in C. glutamicum. The mCherry gene was cloned into the plasmid pZM1, and the expression of mCherry was indicated by the fluorescence intensity at an excitation wavelength of 588 nm and an emission wavelength of 618 nm. Table S1. Plasmids and strains used in the present study. Table S2. Primers used in this study. Table S3. C3G production using concentrated cells in different conditions. Cells grown in AMM (pH7.0) were induced with IPTG (0.5 mM) for 6 h and harvested. Then 5 ml of cells were resuspended in 1 ml of different buffers with 500 mg/L catechin and necessary supplements, including citrate buffer, potassium phosphate buffer, AMM (pH 5.0) and AMM (pH 7.0), respectively. The conversion process was conducted at 30 °C and 220 rpm for 48 h. [file 12934_2018_990_MOESM1_ESM.docx]

**Supplementary Table 1** Plasmids and strains used in the present study.

| **Strain or plasmid** | **Relevant characteristics** | **Source** |
| --- | --- | --- |
| **Strains** |  |  |
| *E. coli* DH5α | F−, φ80d lacZΔM15, Δ(lacZYA-argF)U169, recA1, endA1, hsdR17(rk−, mk+), phoA, supE44λ−, thi−1, gyrA96, relA1 | Novagen |
| *C. glutamicum* ATCC 13032 | Host strain | ATCC |
| **Plasmids** |  |  |
| pEC-XK99E | A *E. coli*-*C. glutamicum* shuttle vector, pGA1 ori, Kan^R^ | [1] |
| pZ8-Ptac | A *E. coli*-*C. glutamicum* shuttle vector, pHM1519 ori, Kan^R^ | Addgene |
| pZ8-1 | A *E. coli*-*C. glutamicum* shuttle vector, pHM1519 ori, Kan^R^ | [2] |
| pCDF-3A | E. coli expression plasmid containing Arabidopsis 3GT fused to N-terminus of Petunia ANS (3A) | [3] |
| pEC-3AO | pEC-XK99E carrying *3A* | This study |
| pZ8-3AO | pZ8-1 carrying *3A* | This study |
| pZM1 | ePathBrick vector features, modified from pZ8-Ptac | This study |
| pEC-o3GT-oANS | Wild-type *3GT* and *ANS* in operon form | This study |
| pEC-oSUMO-3GT-  oANS | *SUMO* tag-fused wild-type *3GT* and *ANS* in operon form | This study |
| pEC-oMBP-3GT-  oANS | *MBP* tag-fused wild-type *3GT* and *ANS* in operon form | This study |
| pEC-o3GT’-oANS’ | Codon-optimized *3GT* and *ANS* in operon form | This study |
| pEC-oSUMO-3GT’-  oANS’ | *SUMO* tag-fused codon- optimized *3GT* and codon-optimized *ANS* in operon form | This study |
| pEC-oMBP-3GT’-  oANS’ | *MBP* tag-fused codon- optimized *3GT* and codon-optimized *ANS* in operon form | This study |
| pEC-m3GT-mANS | Wild-type *3GT* and *ANS* in monocistronic form | This study |
| pEC-mSUMO-3GT-  mANS | *SUMO* tag-fused wild-type *3GT* and wild-type *ANS* in monocistronic form | This study |
| pEC-mMBP3GT-  mANS | *MBP* tag-fused wild-type *3GT* and wild-type *ANS* in monocistronic form | This study |
| pEC-m3GT’-mANS’ | Codon-optimized *3GT* and *ANS* in monocistronic form | This study |
| pEC-mSUMO-3GT’-  mANS’ | *SUMO* tag-fused codon- optimized *3GT* and codon-optimized *ANS* in moncistronic form | This study |
| pEC-mMBP-3GT’-  mANS’ | *MBP* tag-fused codon- optimized *3GT* and codon-optimized *ANS* in monocistronic form | This study |
| pZM1-mSUMO-  3GT-mANS | *SUMO* tag-fused wild-type *3GT* and wild-type *ANS* with monocistronic form in pZM1 | This study |
| pZM1-eftuSUMO-  3GT-eftuANS | *SUMO-3GT* and *ANS* both under the control of the promoter *eftu* in monocistronic form | This study |
| pZM1-eftuSUMO-  3GT-sodANS | *SUMO-3GT* and *ANS* under the control of the promoters *eftu* and *sod* in monocistronic form | This study |
| pZM1-sodSUMO-  3GT-sodANS | *SUMO-3GT* and *ANS* both under the control of the promoter *sod* in monocistronic form | This study |
| pZM1-sodSUMO-  3GT-eftuANS | *SUMO-3GT* and *ANS* under the control of the promoter *sod* and *eftu* separately in monocistronic form | This study |
| pZM1-mPgm-mYcjU-mGalU-mSUMO-3GT-mANS | Expression of *pgm*, *ycjU* and g*alU* from *E. coli*, *SUMO-3GT* and *ANS* in monocistronic form | This study |
| pZM1-mCmk-mNdk-  mPgm-mYcjU-mGalU-mSUMO-3GT-mANS | Expression of *cmk*, *ndk*, *pgm*, *ycjU* and g*alU* from *E. coli*, *SUMO-3GT* and *ANS* in monocistronic form | This study |
| pZM1-mCmk-mNdk- mGalU-mSUMO-  3GT-mANS | Expression of *cmk*, *ndk* and g*alU* from *E. coli*, *SUMO-3GT* and *ANS* in monocistronic form | This study |
| pZM1-mCmk-mNdk-  mPgm-mYcjU-mSUMO-3GT-mANS | Expression of *cmk*, *ndk*, *pgm* and *ycjU* from *E. coli*, *SUMO-3GT* and *ANS* in monocistronic form | This study |
| pZM1-mcgGalU1-  mcgPgm-mSUMO-3GT-mANS | Expression of C. glutamicum-derived *galU1* and *pgm*, *SUMO-3GT* and *ANS* in monocistronic form | This study |
| pZM1-mCsF3H-  mCsDFR-mDuLAR | Expression of *F3H*, *DFR* and *LAR* in monocistronic form | This study |

**Supplementary Table 2** Primers used in this study.

| Primer | Sequence (5’-3’) |
| --- | --- |
| 3GT-F-EcoRI | CCGGAATTC**AAAGGAGGACAACC**ATGACCAAACCCTCCGAC |
| 3GT-R-SalI | ACGCGTCGACGGTACCTCAAATAATGTTTACAACTGCATC |
| ANS-F-KpnI | CGGGGTACC**AAAGGAGGACAACC**ATGGTGAATGCAGTAGTTACAAC |
| ANS-R-PstI | AAAACTGCAGCTATTTAGATTCTTCAGCAGCAA |
| ANS-R-SalI | ACGCGTCGACCTATTTAGATTCTTCAGCAGCAA |
| SUMO-3GT-F1-MfeI | CCGCAATTGGAGCTC**AAAGGAGGACAACC**ATGGGTTCTTCTATGGCTAGCATG |
| SUMO-3GT-R1 | GGAGGGTTTGGTCATTGCATTGGATTGGAAGTACAGGTT |
| SUMO-3GT-F2 | TGTACTTCCAATCCAATGCAATGACCAAACCCTCCGAC |
| SUMO-3GT-R2-kpnI | CGGGGTACCTCAAATAATGTTTACAACTGCATC |
| MBP-3GT-F1-EcoRI | CCGGAATTC**AAAGGAGGACAACC**ATGAAAATCGAAGAAGGTAAACT |
| MBP-3GT-R1 | GGTTTGGTCATCCTTCCCTCGATCCCG |
| MBP-3GT-F2 | GATCGAGGGAAGGATGACCAAACCCTCCGAC |
| MBP-3GT-R2-KpnI | CGGGGTACCTCAAATAATGTTTACAACTGCATC |
| cgANS-F-EcoRI | CCGGAATTCCTAGTCTAGA**AAGGAGGACAACC**ATGGTGAACGCCGTCGT |
| cgANS-R-BamH | CGCGGATCCTTATTACTTGCTTTCTTCAGCCG |
| cg3GT-F-EcoRI | CCGGAATTC**AAAGGAGGACAACC**ATGACCAAGCCGTCAGATCC |
| cg3GT-R-XbaI | CTAGTCTAGATTATTAAATGATGTTCACCACTGCG |
| SUMO-cg3GT-F1-  MfeI | CCGCAATTGCCCGGGAAAGGAGGACAACCATGGGTTCTTC |
| SUMO-cg3GT-R1 | GGCTTGGTCATTGCATTGGATTGGAAGTACAGGTT |
| SUMO-cg3GT-F2 | CAATCCAATGCAATGACCAAGCCGTCAGATCC |
| MBP-cg3GT-F1-  EcoRI | CCGGAATTCAAAGGAGGACAACCATGAAAATCG |
| MBP-cg3GT-R1 | GCTTGGTCATCCTTCCCTCGATCCCGAGG |
| MBP-cg3GT-F2 | GATCGAGGGAAGGATGACCAAGCCGTCAGATCC |
| MBP-cg3GT-R2-XbaI | CTAGTCTAGATTATTAAATGATGTTCACCACTGCG |
| T1T2-Ptac-KpnI-F1 | CGGGGTACCTGCCTGGCGGCAGTAG |
| T1T2-Ptac-R1 | GTCAACAGCTCAAAAGGCCATCCGTCAGG |
| T1T2-Ptac-F2 | GGATGGCCTTTTGAGCTGTTGACAATTAATCATCG |
| T1T2-Ptac-KpnI-R2 | CGGGGTACCGTGTGAAATTGTTATCCGCTCA |
| T1T2-Ptac-XbaI-F1 | CTAGTCTAGATGCCTGGCGGCAGTAG |
| T1T2-Ptac-XbaI-R2 | CTAGTCTAGAGTGAAATTGTTATCCGCTCA |
| EMK-F-NdeI | ACGCCATATGACGGCAATTGCCC |
| EMK-R-BamH | CGCGGATCCTTATGCGAGAGCCAATTTCTG |
| PGM-F-NdeI | ACGCCATATGGCAATCCACAATCGTG |
| PGM-R-BamH | CGCGGATCCTTACGCGTTTTTCAGAACTTCG |
| ycjU-F-NdeI | ACGCCATATGAAACTGCAAGGGGTAAT |
| ycjU-R-BamH | CGCGGATCCCTATACGTTTTGCCAGAAGGC |
| GalU-F-XbaI | CTAGTCTAG**AAAGGAGGACAACC**ATGGCTGCCATTAATACGAAAG |
| GalU-R-BamH | CGCGGATCCTTACTTCTTAATGCCCATCTCTTC |
| NDK-F-NdeI | ACGCCATATGGCTATTGAACGTACTTTTTCC |
| NDK-R-BamH | CGCGGATCCTTAACGGGTGCGCGG |
| ANS-F-AseI | acgcATTAATATGGTGAATGCAGTAGTTACAAC |
| CsF3H-F-NdeI | TATACATATGGCACCGACCACC |
| CsF3H-R-BamHI | CGCGGATCCTTAGGCGAAGATTTCATCGGTG |
| CsDFR-F-NdeI | TATACATATGAAAGATAGCGTTGCA |
| CsDFR-R-BamHI | CGCGGATCCTTACACTTTGTTGCCATTAACCG |
| DuLAR-F-NdeI | TATACATATGACCGTTAGCGGTG |
| DuLAR-R-BamHI | CGCGGATCCTTAACCCATTGCGCTAATCGG |
| eftu-F-XmaJ1 | GAAATCCTAGGTGGCCGTTACCCTGCGAATG |
| eftu-R-NdeI | TATACATATGATGTCCTCCTGGACTTCGTGGTGG |
| sod-F-XmaJ1 | GAAATCCTAGGTAGCTGCCAATTATTCCGGG |
| sod-R-NdeI | TATACATATGTAAAAAATCCTTTCGTAGGTTTCC |
| cgGalU-F-NdeI | TATACATATGAGTTTGCCTATCGATGAGC |
| cgGalU-R-BamHI | CGGGGTACCCTATTTTACTTGAGAATCGTCTGCA |
| cgPGM-F-NdeI | TATACATATGGCACATGAACGCGC |
| cgPGM-R-BamHI | CGGGGTACCTTACTGTCCGAGTACTTCGCTGAC |
|  |  |

Underlined sequences are restriction sites; sequences in bold include ribosome binding sites and spacer sequences applied in this study.

**Supplementary Table 3** C3G production using concentrated cells in different conditions. Cells grown in AMM (pH7.0) were induced with IPTG (0.5 mM) for 6 hours and harvested. Then 5 ml of cells were resuspended in 1 ml of different buffers with 500 mg/L catechin and necessary supplements, including citrate buffer, potassium phosphate buffer, AMM (pH 5.0) and AMM (pH 7.0), respectively. The conversion process was conducted at 30 °C and 220 rpm for 48 h.

| **Conditions** | **C3G Production (mg/L)** |
| --- | --- |
| Citrate Buffer (0.1M, pH 5.0) | 2.45 ± 0.09 |
| AMM (pH 5.0) | 1.26 ± 0.15 |
| Phosphate Buffer (0.1 M, pH 7.0) | 1.58 ± 0.28 |
| AMM (pH 7.0) | 43.67 ± 0.61 |

**Supplementary Figure 1**. SDS-PAGE (10% gel) analysis of proteins ANS and 3GT by recombinant *C. glutamicum* expressing *ANS* and *3GT* in various media. The *E. coli* strain expressing *ANS* and *3GT* was used as a positive control. *E. coli* was cultivated in AMM with 2% glucose and induced by 1 mM IPTG for 4 h before harvested for protein extraction. The *C. glutamicum* strain was grown in BHIS, AMM or CGXII medium and was induced by 1 mM IPTG at mid-exponential phase for 12 h. Red arrows indicate bands for ANS (48.5 kD) and 3GT (50.5 kD) (their molecular weight is too close, and only one merged band could be seen in the gel).


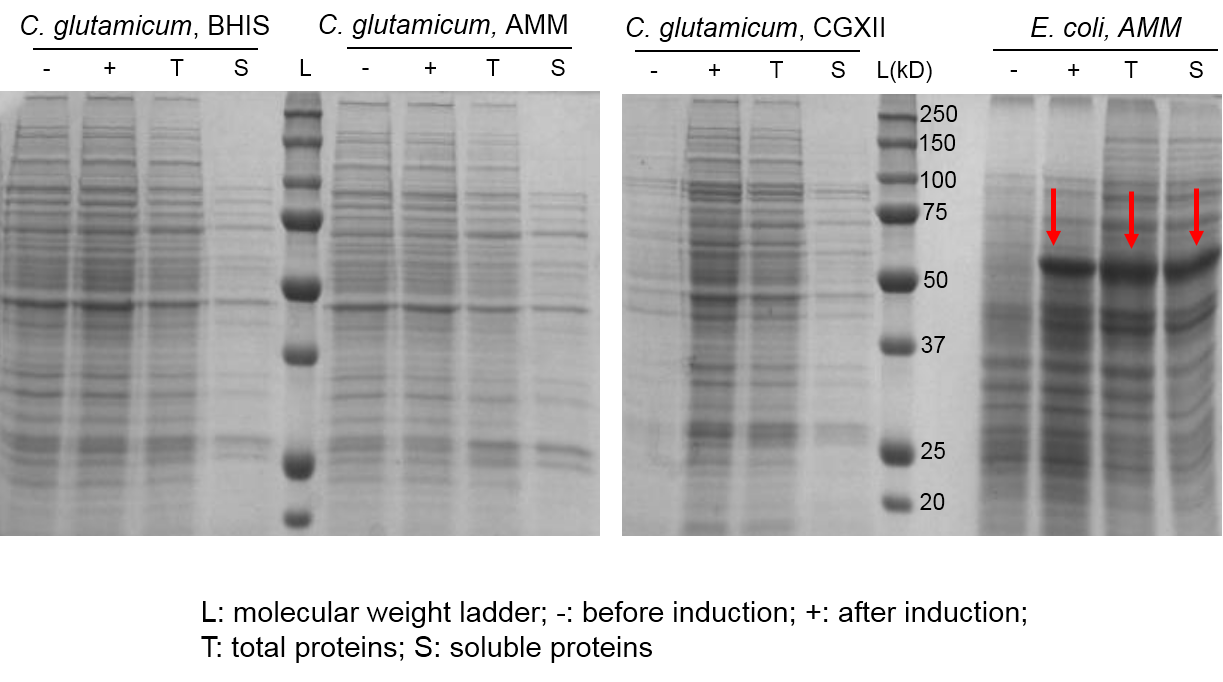


**Supplementary Figure 2**. Mass Spectrum identification of C3G in the fermentation products of recombinant *C. glutamicum* strains. TIC (A) and EIC (B) for mass range of C3G of the standard C3G; TIC(C) and EIC (D)for mass range of C3G for the fermentation products; Mass spectrum for C3G peak in the standard (E) and the fermentation products (F).





**Supplementary Figure 3**. Time course study of mCherry expression in *C. glutamicum*. The mCherry gene was cloned into the plasmid pZM1, and the expression of mCherry was indicated by the fluorescence intensity at an excitation wavelength of 588 nm and an emission wavelength of 618 nm.


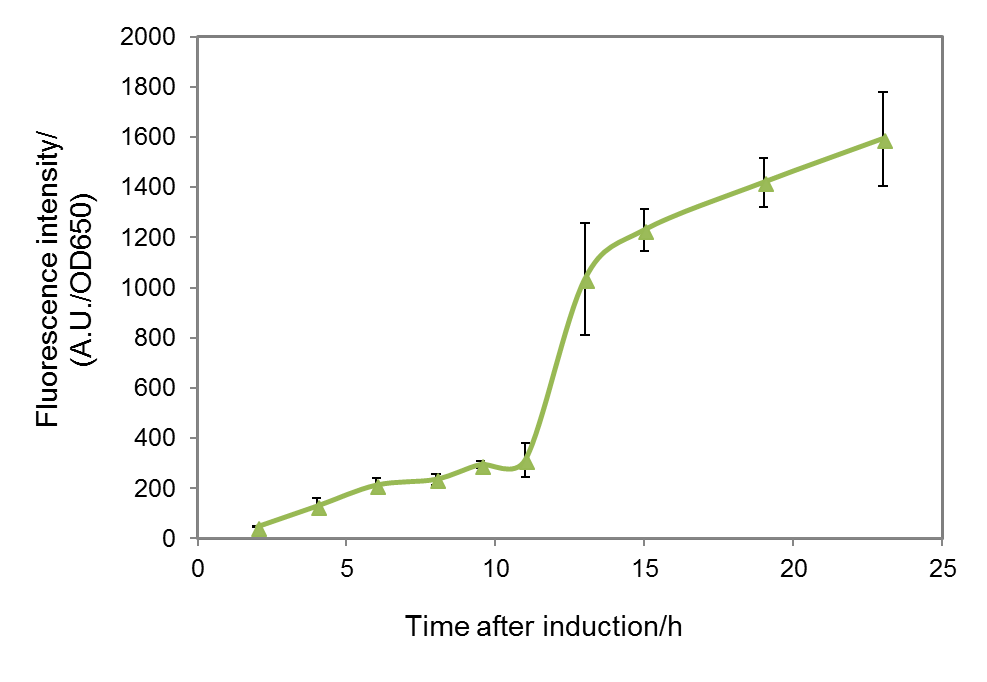


**References**

1. Kirchner O, Tauch A. Tools for genetic engineering in the amino acid-producing bacterium *Corynebacterium glutamicum*. J Biotechnol. 2003;104:287-99.

2. Cleto S, Jensen JVK, Wendisch VF, Lu TK. *Corynebacterium glutamicum* metabolic engineering with CRISPR interference (CRISPRi). ACS Synth Biol. 2016;5:375-85.

3. Yan Y, Li Z, Koffas MA. High-yield anthocyanin biosynthesis in engineered *Escherichia coli*. Biotechnol Bioeng. 2008;100:126-40.
